# Food Insecurity in HIV-Hepatitis C Virus Co-infected Individuals in Canada: The Importance of Co-morbidities

**DOI:** 10.1007/s10461-016-1326-9

**Published:** 2016-02-24

**Authors:** Joseph Cox, Anne-Marie Hamelin, Taylor McLinden, Erica E. M. Moodie, Aranka Anema, Kathleen C. Rollet-Kurhajec, Gilles Paradis, Sean B. Rourke, Sharon L. Walmsley, Marina B. Klein, Canadian Co-infection Cohort Investigators

**Affiliations:** 1 Department of Epidemiology, Biostatistics and Occupational Health, McGill University, Purvis Hall, 1020 Pine Avenue West, Montreal, QC H3A 1A2 Canada; 2 Chronic Viral Illness Service, McGill University Health Centre, Montreal, QC Canada; 3 CIHR Canadian HIV Trials Network, Vancouver, BC Canada; 4 Department of Pediatrics, Harvard Medical School, Boston, MA USA; 5 Department of Medicine, Boston Children’s Hospital, Boston, MA USA; 6 Li Ka Shing Knowledge Institute, St. Michael’s Hospital, Toronto, ON Canada; 7 The Ontario HIV Treatment Network, Toronto, ON Canada; 8 Department of Psychiatry, University of Toronto, Toronto, ON Canada; 9 Department of Medicine, University of Toronto, Toronto, ON Canada; 10 Division of Infectious Diseases, University Health Network, Toronto, ON Canada

**Keywords:** Food insecurity, HIV, HCV, Co-infection, Canada

## Abstract

While research has begun addressing food insecurity (FI) in HIV-positive populations, knowledge regarding FI among individuals living with HIV-hepatitis C virus (HCV) co-infection is limited. This exploratory study examines sociodemographic, socioeconomic, behavioral, and clinical factors associated with FI in a cohort of HIV-HCV co-infected individuals in Canada. We analyzed longitudinal data from the Food Security and HIV-HCV Co-infection Study of the Canadian Co-infection Cohort collected between November 2012-June 2014 at 15 health centres. FI was measured using the Household Food Security Survey Module and classified using Health Canada criteria. Generalized estimating equations were used to assess factors associated with FI. Among 525 participants, 59 % experienced FI at their first study visit (baseline). Protective factors associated with FI (*p* < 0.05) included: enrolment at a Quebec study site (aOR: 0.42, 95 % CI: 0.27, 0.67), employment (aOR: 0.55, 95 % CI: 0.35, 0.87), and average personal monthly income (aOR per $100 CAD increase: 0.98, 95 % CI: 0.97, 0.99). Risk factors for FI included: recent injection drug use (aOR: 1.98, 95 % CI: 1.33, 2.96), trading away food (aOR: 5.23, 95 % CI: 2.53, 10.81), and recent experiences of depressive symptoms (aOR: 2.11, 95 % CI: 1.48, 3.01). FI is common in this co-infected population. Engagement of co-infected individuals in substance use treatments, harm reduction programs, and mental health services may mitigate FI in this vulnerable subset of the HIV-positive population.

**Electronic supplementary material:**

The online version of this article (doi:10.1007/s10461-016-1326-9) contains supplementary material, which is available to authorized users.

## Introduction

Food insecurity (FI) is an important issue in HIV-positive populations [1–4]. FI exists “whenever the availability of nutritionally adequate and safe foods or the ability to acquire acceptable foods in socially acceptable ways is limited or uncertain” (e.g., without resorting to emergency food supplies, scavenging, stealing, and other coping strategies) [5]. A British Columbia study found that FI among individuals living with HIV exceeded 70 % in 2007–2008, an increase of over 23 % from 1998–1999 [1, 4] when measured using the Radimer/Cornell Questionnaire [6]. Similarly, the prevalence of FI among HIV-positive individuals in Ontario was recently found to be 69 % when measured by the Household Food Security Survey Module (HFSSM) [7]. This is in contrast to the annual estimate of 8 % of Canadian adults who experienced FI between 2007 and 2012 [8].

In HIV-positive populations in Canada and the United States, FI has been associated with sub-optimal combination antiretroviral treatment (cART) adherence [9, 10] and numerous adverse health outcomes, such as: incomplete HIV viral load suppression [11], lower CD4 cell counts [3, 4], and higher rates of mortality [12, 13]. Many pathways have been proposed for FI’s affect on these outcomes. For example, it has been suggested that fear or the actual experience of the side effects of cART are exacerbated in the absence of food, affecting treatment adherence [10]. Additionally, biologic mechanisms such as the impact of food on cART pharmacokinetics [11] and subsequent HIV viral load suppression may have a role. Lastly, nutritional deficiencies are associated with immunosuppression and lower CD4 cell counts in individuals experiencing FI [14].

Due to these adverse outcomes, research in the HIV-setting has identified a variety of risk factors for FI, including: younger age, unstable housing, unemployment, low income, illicit drug use, and experiences of depressive symptoms [15–17]. Indeed, the relationship between FI and HIV has been described as a ‘vicious cycle’ [15, 17] whereby sociodemographic, socioeconomic, behavioral, and clinical risk factors act as drivers of FI, putting individuals at higher risk for adverse health outcomes. These outcomes ultimately affect an individual’s ability to acquire food, thereby perpetuating FI [18].

In the aforementioned research involving HIV-positive populations [1, 4, 7], an unknown proportion of study participants may have been co-infected with hepatitis C virus (HCV). For that reason, findings from these studies may be not generalizable to HIV-HCV co-infected populations and knowledge regarding FI, including estimates of the prevalence and severity of FI, is limited in populations that are known to be living with both viral infections. In Canada, 20 % of the HIV-positive population is estimated to be HIV-HCV co-infected [19] and these individuals are a vulnerable subset of this population. While cART has made HIV infection a chronic manageable condition, co-morbidities such as liver disease and liver-related mortality are increasing [20]. A recent study estimated that all-cause mortality in a Canadian population of co-infected individuals is 12 times higher than that of the overall Canadian population of similar sex and age; liver disease and drug overdose were among the most frequent causes of death [21].

Research comparing HIV mono-infected and co-infected individuals has consistently highlighted the cumulative vulnerabilities experienced in co-infection, many of which may act on one’s ability to acquire food. Studies suggest that the sociodemographic (e.g., unstable housing), socioeconomic (e.g., unemployment), behavioral (e.g., injection drug use), and clinical (e.g., depressive symptoms) characteristics of this subpopulation lead to more fatigue, poorer quality of life, and less social support [22, 23]. It has also been shown that co-infected individuals experience more co-morbid conditions [24] and a Canadian study has documented differences in the social determinants of health between mono-infected and co-infected individuals [25]. Despite HIV-HCV co-infected individuals being an important HIV-positive subpopulation, where we hypothesize that FI may be highly prevalent and more severe, this issue has not been studied in a population of HIV-positive individuals known to be co-infected with HCV.

This exploratory study was conducted to identify sociodemographic, socioeconomic, behavioral, and clinical factors associated with FI in a population of HIV-HCV co-infected individuals in Canada. While this hypothesis-generating work provides insights into factors that may be targeted for potential intervention, the primary objective was to suggest important variables for consideration in future hypothesis-confirming analyses.

## Methods

### Study Design and Population

The Canadian Co-infection Cohort (CCC) is a prospective multi-centre study recruiting HIV-HCV co-infected individuals who receive care from urban and semi-urban HIV clinics in Canada [26]. This study, initiated in 2003, follows participants in 6 Canadian provinces (British Columbia, Ontario, Quebec, Alberta, Saskatchewan, and Nova Scotia); participating health centres routinely screen all HIV-positive individuals for HCV infection. The eligibility criteria for the CCC are: older than 16 years of age, documented HIV infection (HIV seropositive by ELISA with Western blot confirmation), and evidence of HCV infection (HCV RNA positive and/or HCV seropositive by ELISA with RIBA II, EIA confirmation) [26]. All eligible individuals are invited to participate in the CCC where longitudinal data collection (questionnaires and blood samples) occurs every 6 months.

In 2012, a mixed methods Food Security and HIV-HCV Co-infection Study (FS Study) was initiated within the CCC and data collection related to FI was integrated into CCC study visits (i.e., an additional questionnaire was administered by a study coordinator during routine clinical visits). All CCC participants were invited to enroll in the FS Study. Between November 2012 and June 2014, 525 co-infected participants completed at least one FI assessment at 15 study sites, with 274 completing a second assessment, 21 a third, and 1 a fourth; these individuals make up our study sample. Non-participation, formal withdrawals, deaths, and losses to follow-up in the FS Study were recorded at each site. The FS Study and CCC were approved by the research ethics boards of all participating institutions [26] and the community advisory committee of the CIHR Canadian HIV Trials Network.

### Measures

#### Food Insecurity Assessment

At each study visit, FI in the past 6 months was measured using the 10-item adult scale of the HFSSM [27]. Modification of the HFSSM to a shorter reference period, as done in this study (i.e., from 12 to 6 months), has been justified in previous literature [28]. The HFSSM focuses on self-reports of uncertain, insufficient or inadequate food access, availability and utilization due to limited financial resources, and the compromised eating patterns and food consumption that may result.

Health Canada categorizes FI as food secure (none, or one, indication of difficulty with income-related food access), moderate FI (indication of compromise in quality and/or quantity of food consumed), and severe FI (multiple indications of reduced food intake and disrupted eating patterns) [27]. The HFSSM was adapted from the Current Population Survey Food Security Supplement that has been administered in the United States since 1995 [28]; the HFSSM differs primarily in the FI category labels and the thresholds for defining severity categories [27]. Health Canada categorizes FI according to the number of affirmative responses on the 10-item HFSSM [27]. For example, one item asks: “you and other household members worried that food would run out before you got money to buy more. Was that often true, sometimes true, or never true?” The affirmative responses “often true” and “sometimes true” are treated equally. While previous work acknowledges that the tool does not differentiate frequency-of-occurrence information, it has been shown that either response is indicative of FI [6]. Each of the 10 items used to measure FI and the baseline frequency of responses are described in Table S1 (Supplementary Material). All 10 items, regardless of the severity of FI, are treated equally, where it has been shown that more severe items (i.e., items 6–10) are less frequently affirmed than less severe items [29]. To assess the internal consistency of the HFSSM over time, Cronbach’s alpha were calculated at each study visit.

As per Health Canada criteria, participants with 0–1, 2–5, or >6 affirmative responses were classified respectively as being food secure, moderately food insecure, or severely food insecure [27]. For regression modeling, we defined the outcome as a binary indicator of FI (food secure vs. food insecure), where participants with >2 affirmative responses were classified as food insecure (collapsing moderate and severe FI into a single category) in the past 6 months.

#### Covariates Describing the Study Sample

In addition to the covariates evaluated for their association with FI, the following factors (some of which are possible consequences of FI and therefore not included in regression models) were used to describe the study sample at baseline (Table 1). Behavioral factors included: use of food assistance (yes vs. no) and doing the following for food: borrowing money, stealing, begging, selling or pawning personal or household items, delaying paying rent or bills (yes vs. no). Clinical factors included: HIV and HCV infection duration (years), CD4 count (cells/µL), HIV RNA (<50 copies/mL; yes vs. no), body mass index (kg/m^2^), diagnoses of end-stage liver disease or AIDS-defining illnesses (yes vs. no), HCV treatment naïve (yes vs. no), missing cART doses in the past 4 days (yes vs. no), and healthcare usage (emergency room visit or hospitalization; yes vs. no).

**Table 1:** Baseline descriptive characteristics and factors evaluated for their association with food insecurity in 525 HIV-HCV co-infected study participants between November 2012 and June 2014, Canada

Characteristics	Total (N = 525)	Food secure (N = 213)	Food insecure (N = 312)			Missing obs.^a^
			Moderate (N = 129)	Severe (N = 183)	Total (N = 312)	
Sociodemographic	Values are number of participants (%) or median (Q1, Q3)					
Age (years)	49.1 (43.5, 54.1)	50.4 (45.1, 55.2)	48.1 (41.1, 52.9)	48.5 (43.7, 53.5)	48.4 (42.7, 53.5)	0
Male	390 (74 %)	170 (80 %)	90 (70 %)	130 (71 %)	220 (71 %)	0
Born in Canada	395 (75 %)	162 (76 %)	97 (75 %)	136 (74 %)	233 (75 %)	88
Province of enrolment	–	–	–	–	–	–
British Columbia	171 (33 %)	50 (24 %)	50 (39 %)	71 (39 %)	121 (39 %)	0
Ontario	95 (18 %)	41 (19 %)	27 (21 %)	27 (15 %)	54 (17 %)	0
Quebec	248 (47 %)	115 (54 %)	48 (37 %)	85 (46 %)	133 (43 %)	0
Other^b^	11 (2 %)	7 (3 %)	4 (3 %)	0	4 (1 %)	0
Aboriginal	78 (15 %)	24 (11 %)	28 (22 %)	26 (14 %)	54 (17 %)	9
Heterosexual	364 (69 %)	138 (65 %)	96 (74 %)	130 (71 %)	226 (72 %)	0
Living alone^c^	274 (52 %)	103 (48 %)	74 (57 %)	97 (53 %)	171 (55 %)	0
Without a fixed address^c^	23 (4 %)	3 (1 %)	4 (3 %)	16 (9 %)	20 (6 %)	0
Recent changes in housing situation^d^	140 (27 %)	46 (22 %)	32 (25 %)	62 (34 %)	94 (30 %)	0
Recent incarceration^d^	55 (10 %)	15 (7 %)	14 (11 %)	26 (14 %)	40 (13 %)	0
Socioeconomic						
Employment^c^	97 (19 %)	64 (30 %)	21 (16 %)	12 (7 %)	33 (11 %)	0
Average personal monthly income ($ CAD)^d^	1015 (918, 1400)	1111 (934, 2500)	1100 (918, 1300)	966 (897, 1100)	1000 (916, 1200)	3
More than high school education	132 (25 %)	75 (35 %)	21 (16 %)	36 (20 %)	57 (18 %)	0
Behavioral						
Recent injection drug use^d^	180 (34 %)	44 (21 %)	48 (37 %)	88 (48 %)	136 (44 %)	0
Marijuana use^c^	287 (55 %)	94 (44 %)	68 (53 %)	125 (68 %)	193 (62 %)	0
Cigarette use^c^	372 (71 %)	132 (62 %)	96 (74 %)	144 (79 %)	240 (77 %)	46
Alcohol use^c^	306 (58 %)	115 (54 %)	74 (57 %)	117 (64 %)	191 (61 %)	45
>5 alcoholic drinks per day^c^	96 (18 %)	21 (10 %)	26 (20 %)	49 (27 %)	75 (24 %)	48
Trading away food^d,e^	75 (14 %)	6 (3 %)	18 (14 %)	51 (28 %)	69 (22 %)	0
Use of food assistance (in the past month)	346 (66 %)	82 (38 %)	109 (84 %)	155 (85 %)	264 (85 %)	0
Doing the following for food^d^	–	–	–	–	–	–
Borrowing money	224 (43 %)	25 (12 %)	65 (50 %)	134 (73 %)	199 (64 %)	0
Going through garbage	25 (5 %)	1 (<1 %)	5 (4 %)	19 (10 %)	24 (8 %)	0
Stealing	64 (12 %)	4 (2 %)	13 (10 %)	47 (26 %)	60 (19 %)	0
Begging	60 (11 %)	3 (1 %)	9 (7 %)	48 (26 %)	57 (18 %)	0
Selling or trading items	104 (20 %)	5 (2 %)	23 (18 %)	76 (42 %)	99 (32 %)	0
Having sex for food	22 (4 %)	2 (1 %)	4 (3 %)	16 (9 %)	20 (6 %)	0
Trading substances	52 (10 %)	3 (1 %)	11 (9 %)	38 (21 %)	49 (16 %)	0
Delaying payments	115 (22 %)	7 (3 %)	22 (17 %)	86 (47 %)	108 (35 %)	0
Clinical						
HIV infection duration (years)	15.5 (10.3, 20.7)	16.7 (10.4, 22.3)	14.5 (9.9, 19.9)	15.2 (10.3, 19.3)	15.0 (10.3, 19.4)	23
HCV infection duration (years)	23.1 (15.7, 30.7)	22.9 (11.2, 30.1)	22.0 (16.9, 31.2)	24.0 (17.2, 30.7)	23.3 (17.1, 31.0)	0
CD4 count (cells/µL)	460 (310, 680)	493.5 (320, 705)	466 (314, 692)	432 (300, 620)	446 (300, 632)	7
HIV RNA (<50 copies/mL)	412 (78 %)	172 (81 %)	105 (81 %)	135 (74 %)	240 (77 %)	16
Body mass index (kg/m^2^)	23.7 (21.2, 27.2)	24.1 (21.4, 27.6)	24.2 (22.1, 27.4)	23.3 (20.7, 26.6)	23.6 (21.1, 27.0)	67
End-stage liver disease diagnosis^f^	82 (16 %)	39 (18 %)	13 (10 %)	30 (16 %)	43 (14 %)	0
AIDS-defining illness diagnosis^f^	146 (28 %)	66 (31 %)	33 (26 %)	47 (26 %)	80 (26 %)	0
HCV treatment naïve^f^	331 (63 %)	113 (53 %)	93 (72 %)	125 (68 %)	218 (70 %)	0
Taking cART^c^	484 (92 %)	201 (94 %)	123 (95 %)	160 (87 %)	283 (91 %)	0
Missing cART doses (in the past 4 days)	110 (21 %)	31 (15 %)	28 (22 %)	51 (28 %)	79 (25 %)	0
Self-described health state—visual analogue scale (0–100)^c^	70 (55, 80)	75 (60, 85)	70 (50, 80)	65 (50, 75)	70 (50, 80)	4
Therapy for drug addiction^d^	97 (19 %)	27 (13 %)	21 (16 %)	49 (27 %)	70 (22 %)	29
Recent depressive symptoms (CES-D-10) (in the past week)	276 (53 %)	76 (36 %)	67 (52 %)	133 (73 %)	200 (64 %)	13
Unmet healthcare needs^d^	95 (18 %)	19 (9 %)	26 (20 %)	50 (27 %)	76 (24 %)	0
Healthcare usage^d^	–	–	–	–	–	–
Emergency room visit	144 (27 %)	48 (23 %)	40 (31 %)	56 (31 %)	96 (31 %)	0
Hospitalization	75 (14 %)	23 (11 %)	20 (16 %)	32 (17 %)	52 (17 %)	0

^a^Number of observations missing at the baseline assessment

^b^Provinces of Alberta, Saskatchewan, and Nova Scotia

^c^Reference period: currently

^d^Reference period: in the past 6 months

^e^Trading away food for: tobacco, personal or household items, drugs, alcohol, sex, a place to stay

^f^Reference period: lifetime

#### Covariates Evaluated for Their Association with Food Insecurity

Informed by existing evidence regarding FI in HIV-positive populations in Canada and the United States [15–17], various sociodemographic, socioeconomic, behavioral, and clinical factors were evaluated for their potential association with FI (Table 2). Sociodemographic factors included: age (years), sex (male vs. female), born in Canada (yes vs. no), province of study enrolment (Ontario, Quebec, Other [Alberta, Saskatchewan, Nova Scotia] vs. British Columbia), Aboriginal (First Nations, Inuit, or Métis vs. Other), sexual orientation (heterosexual vs. homosexual or bisexual), living alone (yes vs. no), without a fixed address (yes vs. no), recent changes in housing situation (yes vs. no), and recent incarceration (yes vs. no). Socioeconomic factors included: employment (yes vs. no), average personal monthly income (including all sources, before taxes and deductions; $ CAD: Canadian dollars), and more than high school education (yes vs. no). Behavioral factors included: substance use (i.e., marijuana, cigarettes, alcohol, injection drugs; yes vs. no), trading away food for: tobacco, personal or household items, drugs, alcohol, sex, a place to stay (yes vs. no). Clinical factors included: taking cART (yes vs. no), self-described health state (visual analogue scale, 0 = worst imaginable health state to 100 = best imaginable health state), recent therapy for drug addiction (yes vs. no), experiences of recent depressive symptoms (measured using the 10-item Center for Epidemiological Studies Depression Scale: CES-D-10, and defined by a score of >10 on the scale; yes vs. no) [30], and self-reports of unmet healthcare needs (yes vs. no).

**Table 2:** Univariate and multivariate logistic regression models of factors associated with food insecurity in 525 HIV-HCV co-infected study participants between November 2012 and June 2014, Canada

	Univariate OR (95 % CI)^a^	*p* value^b^	Multivariate aOR (95 % CI)	*p* value
Sociodemographic				
Intercept	–	–	4.78 (0.98, 23.41)	0.054
Age: per 5-year increase	0.85 (0.77, 0.94)	<0.001	0.90 (0.81, 1.00)	0.057
Male	1.58 (1.07, 2.31)	0.020	1.26 (0.78, 2.04)	0.343
Born in Canada	1.09 (0.62, 1.91)	0.764	–	–
Province of enrolment	–	–	–	–
British Columbia	Ref.	Ref.	Ref.	Ref.
Ontario	0.50 (0.31, 0.81)	0.005	0.74 (0.43, 1.27)	0.275
Quebec	0.45 (0.30, 0.65)	<0.001	0.42 (0.27, 0.67)	<0.001^*^
Other^c^	0.24 (0.07, 0.84)	0.025	0.49 (0.14, 1.73)	0.265
Aboriginal	1.75 (1.08, 2.82)	0.022	1.12 (0.58, 2.13)	0.759
Heterosexual	1.52 (1.07, 2.16)	0.019	1.12 (0.73, 1.70)	0.605
Living alone^d^	1.44 (1.09, 1.89)	0.010	1.31 (0.92, 1.87)	0.128
Without a fixed address^d^	2.19 (1.16, 4.14)	0.016	1.19 (0.50, 2.84)	0.689
Recent changes in housing situation^e^	1.44 (1.06, 1.95)	0.021	1.11 (0.73, 1.68)	0.635
Recent incarceration^e^	1.96 (1.14, 3.36)	0.015	1.11 (0.59, 2.08)	0.749
Socioeconomic				
Employment^d^	0.34 (0.24, 0.49)	<0.001	0.55 (0.35, 0.87)	0.010^*^
Average personal monthly income ($ CAD): per $100 increase^e^	0.96 (0.93, 0.99)	0.012	0.98 (0.97, 0.99)	0.001^*^
More than high school education	0.45 (0.30, 0.66)	<0.001	0.72 (0.45, 1.13)	0.155
Behavioral				
Recent injection drug use^e^	2.79 (2.02, 3.85)	<0.001	1.98 (1.33, 2.96)	<0.001^*^
Marijuana use^d^	1.82 (1.37, 2.42)	<0.001	1.41 (0.99, 2.01)	0.060
Cigarette use^d^	1.67 (1.18, 2.35)	0.004	0.98 (0.65, 1.50)	0.940
Alcohol use^d^	1.28 (0.97, 1.67)	0.077	1.21 (0.84, 1.75)	0.301
>5 alcoholic drinks per day^d^	1.55 (1.06, 2.25)	0.023	1.21 (0.75, 1.94)	0.433
Trading away food^e,f^	5.99 (3.53, 10.16)	<0.001	5.23 (2.53, 10.81)	<0.001^*^
Clinical				
Taking cART^d^	0.52 (0.31, 0.88)	0.015	0.68 (0.36, 1.29)	0.237
Self-described health state—visual analogue scale (0–100): per 5-point increase^d^	0.93 (0.89, 0.96)	<0.001	0.98 (0.94, 1.02)	0.242
Therapy for drug addiction^e^	1.16 (0.79, 1.71)	0.452	–	–
Recent depressive symptoms (CES-D-10) (in the past week)	2.78 (2.06, 3.74)	<0.001	2.11 (1.48, 3.01)	<0.001^*^
Unmet healthcare needs^e^	2.25 (1.55, 3.26)	<0.001	1.55 (0.93, 2.57)	0.091

^a^ *OR* odds ratios, 95 % *CI* confidence intervals, and *aOR* adjusted ORs estimated from logistic regression models (outcome: food secure vs. food insecure) using GEE

^b^Factors *p* < 0.1 in univariate models were included in the multivariate model

^c^Provinces of Alberta, Saskatchewan, and Nova Scotia

^d^Reference period: currently

^e^Reference period: in the past 6 months

^f^Trading away food for: tobacco, personal or household items, drugs, alcohol, sex, a place to stay

* *p* < 0.05

The reference periods for each measurement are indicated in the table footnotes. The following factors were ascertained by research nurses/coordinators at study sites: CD4 count (cells/µL), HIV RNA (<50 copies/mL), body mass index (kg/m^2^), diagnoses of end-stage liver disease or AIDS-defining illnesses, HCV treatment naïve, and taking cART. All other factors were self-reported by participants.

### Data Analyses

Summary statistics were used to describe the study sample at baseline and frequencies were stratified by FI and FI severity: food secure, moderate FI, and severe FI. Multiple imputation by chained equations were used to impute missing observations across all study visits using 20 imputations and 25 iterations, with continuous variables imputed using predictive mean matching and logistic regression (including polytomous regression) used for categorical variables. To account for repeated measurements on participants over time, generalized estimating equations (GEE) were used to estimate the marginal parameters of univariate and multivariate logistic regression models with a working autoregressive correlation structure.

The dependent variable was a binary indicator of FI (food secure vs. food insecure), collapsing moderate and severe FI into a single category. Factors evaluated for their association with FI were included in the logistic models as independent variables. Given the exploratory nature of the study, all factors significant at the liberal two-tailed *p* < 0.1 in univariate models were simultaneously included in the multivariate model; no model reduction was performed. Statistical significance in the multivariate model was defined as a two-tailed *p* < 0.05. All models were fit to each of the 20 imputed datasets, where the coefficients and variance estimates were subsequently combined using Rubin’s method to account for between-imputation variability [31]. All data analyses were performed using R (Version 3.2.0—R Foundation for Statistical Computing).

## Results

Due to the number of participants with only a single baseline assessment (N = 251 of 525, 48 %), the mean follow-up time in the FS Study was 0.20 years (IQR: 0.0, 0.47). Between November 2012 and June 2014, 9 participants refused to enroll in the study, 0 withdrew, 14 died, and 0 participants were lost to follow-up (based on the CCC definition for loss to follow-up, missing 3 consecutive study visits over 18 months). The internal consistency of the 10-item HFSSM was acceptable in this study sample [32], where Cronbach’s alpha at each study visit exceeded 0.90.

Table 1 indicates that the majority of the participants were food insecure (N = 312, 59 %); 41 and 59 % of these participants experienced moderate and severe FI, respectively. The median age of the study sample was 49.1 years (IQR: 43.5, 54.1), 74 % were male, and 47 % of participants were enrolled at a Quebec study site. A small proportion of the sample did not have a fixed address (4 %). Nineteen percent of participants were employed, and the average personal monthly income was $1015 CAD (IQR: 918, 1400). The majority of the participants engaged in cigarette (71 %), alcohol (58 %), and marijuana (55 %) use, and recently experienced depressive symptoms (53 %). Also, 92 % of participants were taking cART for HIV and 34 % reported use of injection drugs in the past 6 months. Borrowing money to buy food was a common strategy used by food insecure participants. Lastly, a notable proportion of participants experiencing FI traded away food (22 %), most commonly for tobacco, personal or household items, or drugs.

Table 2 indicates that all but two factors (i.e., whether or not the participant was born in Canada and whether or not the participant had recently been in therapy for drug addiction) were significantly associated with FI in univariate logistic regressions. In the multivariate model, protective factors that remained significantly associated with FI (*p* < 0.05) included: enrolment at a Quebec study site (aOR: 0.42, 95 % CI: 0.27, 0.67), employment (aOR: 0.55, 95 % CI: 0.35, 0.87), and average personal monthly income (aOR per $100 CAD increase: 0.98, 95 % CI: 0.97, 0.99). Significant risk factors for FI included: recent injection drug use (aOR: 1.98, 95 % CI: 1.33, 2.96), trading away food (aOR: 5.23, 95 % CI: 2.53, 10.81), and recent experiences depressive symptoms (aOR: 2.11, 95 % CI: 1.48, 3.01).

## Discussion

FI is common (59 %) in this co-infected population and the majority of food insecure participants experienced severe FI, indicating reduced food intake and disrupted eating patterns [27]. In previous evaluations of HIV-positive populations in British Columbia and Ontario, approximately 70 % of participants were food insecure [1, 7]; FI severity was not explored in these studies. However, these single-province prevalence estimates may not be directly comparable as our interprovincial study uses a different FI assessment tool than the British Columba study.

Geographically, most Canadian FI-related research involving HIV-positive populations has been conducted in British Columbia [1, 4]. In our study, important differences in the proportions of individuals experiencing FI were noted across provinces, where the baseline proportions of participants experiencing FI in British Columbia, Ontario, and Quebec were 71, 57, and 54 %, respectively. Therefore, the prevalence of FI in our co-infected study sample in British Columbia is similar to that documented in an HIV-positive population in this province (71 %) [1]. However, given our use of interprovincial data (approximately half of the study sample is from Quebec; FI prevalence of 54 %); the estimated prevalence of FI (59 %) is reduced compared to the British Columbia study. Additionally, this study used a different FI assessment tool (Radimer/Cornell questionnaire vs. HFSSM) with different thresholds to define FI [29]. Lastly, as described, an unknown proportion of HIV-positive participants in the British Columbia study may have been co-infected with HCV, making direct comparisons of FI prevalence difficult. Regardless of these differences, these prevalence estimates consistently indicate high levels of deprivation in terms of food access among these populations; they are markedly higher than the annual estimate of 8 % for Canadian adults [8].

As described, co-infected populations experience less favourable sociodemographic, socioeconomic, behavioral, and clinical conditions leading to more fatigue, poorer quality of life, less social support, and more co-morbid conditions [23–25]. These cumulative vulnerabilities may modify the effects of risk factors for FI in the co-infection context. For example, recent experiences of depressive symptoms, identified as a significant risk factor in both our study sample as well as in HIV-positive populations, may have a stronger effect in co-infected individuals given cumulative vulnerabilities (e.g., less social support, more substance use, and other co-morbidities). In order to investigate this hypothesis, one could compare the effects estimated in our study to those found in studies of HIV mono-infected populations in similar settings. However, given that previous studies in Canada do not report on the presence of HCV co-infection, such comparisons are challenging. Alternatively, a quantitative assessment of this hypothesis would require a study population containing both HIV mono-infected and HIV-HCV co-infected individuals, and where co-infection status is known. In such a scenario, effect measure modification could be assessed in regression modeling by creating an interaction term between an indicator for being co-infected and the risk factor(s) of interest. This is not possible in our study as all participants are co-infected with HCV. Therefore, we recommend that future research report on the presence of HCV co-infection and explore whether the cumulative vulnerabilities experienced in co-infection modify the effects of risk factors for FI.

Regarding protective factors in the multivariate model, Quebec participants were less likely to experience FI (using British Columbia as the referent province) after adjustment for many sociodemographic, socioeconomic, behavioral, and clinical factors. However, the underlying reasons for provincial differences in FI in our study, including differences in the severity of FI, remain to be explained. Qualitative interview data on FI, collected among FS Study participants in Vancouver, Toronto, and Montreal may provide useful insights. Also, future analyses will use additional data from the FS Study to examine the distributions of important contextual factors (e.g., social support services) across provinces and describe their effects on FI in these distinct geographical regions.

Recognizing that direct comparisons with past studies are difficult, many of the other observed associations are consistent with previous studies in HIV-positive populations [1–4]. Both greater average personal monthly income and current employment were significantly and negatively associated with FI. These associations were expected given the HFSSMs focus on inadequate food access due to limited financial resources [27]. Regarding risk factors for FI, 34 % of participants reported injecting drugs in the past 6 months and recent injection drug use was significantly associated with FI. Illicit drug use (including but not limited to injection drug use) has previously been described as a risk factor for FI in HIV-positive populations [1–4]. Illicit drug use is believed to act on FI through behavioral and environmental pathways [16] and may contribute to FI by further disrupting food intake patterns, resulting in the consumption of foods that are inadequate in quantity and/or quality [33, 34]. Illicit drug using environments also contribute to FI by imposing social, economic, physical, and policy barriers to food access and availability [35–39]. As suggested by others [1–4], our work also indicates that FI is an associated harm of injection drug use. Therefore, engagement of co-infected individuals in substance use treatments and harm reduction programs (e.g., opioid substitution programs and other clinical services used by individuals who inject drugs) may act to mitigate FI in this population. However, it should be noted that the unadjusted effect of recent drug addiction therapy on FI was non-significant. This contradiction may be explained by the fact that this estimate reflects the effect of being in recent therapy (in the past 6 months). It is possible that those in recent therapy have yet to experience the benefits of such programs. Also, if the individuals engaging in therapy are those with the highest frequency and duration of substance use, this unadjusted effect is likely to be confounded by such factors. Lastly, addiction therapy is only one treatment program available for individuals who use drugs. We suspect that in addition to addiction therapy, other clinical services and harm reduction programs are necessary for reducing FI. Future hypothesis-confirming research will use effect decomposition and mediation analyses, where modeling is guided by directed acyclic graphs, to better understand the pathways underlying injection drug use, drug addiction, and FI.

With the exception of injection drug use, none of the other substance use variables (use of marijuana, cigarettes, or alcohol) remained significant in the adjusted model. This may be partially explained by a trajectory of drug use [40] wherein injection often occurs after an individual builds a tolerance to, and becomes dependent on, a highly addictive substance (e.g., opiates, cocaine) [41]. Therefore, while the instability of injecting drugs may significantly affect an individual’s FI [42], this may be less so for other substances, given differences in mechanisms of action, effects, and potential harms. However, the non-significant effects for these substances may be due to different reference periods for use and no information on the frequency and duration of use. These are important considerations as a recent study in an HIV-positive population in the United States found that those who use tobacco and alcohol may use up to one-quarter of their financial resources on these substances [43]. As shown in Table 1, the majority of food insecure participants used marijuana, cigarettes, and also consumed alcohol. Therefore, in a co-infected population where substance use is common, sufficient details regarding the use of all substances (including illicit use by non-injection), as well as how priorities are established in the context of limited resources, are needed to understand their roles in contributing to FI.

Competing needs and demands on financial resources are an issue in this HIV-HCV co-infected population. The largest effect on FI was observed for trading food, where trading food for tobacco, personal or household items, or drugs were the most common practices. Research has documented that even in situations of hunger, individuals may use food as a commodity to meet other needs. In studies of street-involved youth and inner city drug-using women, food is treated as a coveted resource that can be used to gain favour, including drugs [44–46]. A possible explanation is that food can be obtained for free from distribution sites. Therefore, in the context of substance use and addiction, food may be forgone or traded to meet other needs, potentially leading to FI.

Similarly to the study of FI in an HIV-positive population in British Columbia [1], our work demonstrates that recent experiences of depressive symptoms are a significant risk factor for FI. Different mechanisms have been proposed, including the role of neurovegetative symptoms: loss of interest, appetite change, psychomotor agitation or retardation, fatigue, and a diminished ability to think or concentrate [47]. Alternatively, experiencing FI implies uncertainty about food access [27], possibly inciting coping strategies (e.g., obtaining food in socially unacceptable ways) that may lead to stress and depression in the long term [48, 49]. Although directionality is unclear, the experience of depressive symptoms is linked with the experience of FI. As such, FI in co-infected individuals may be mitigated through the use of mental health services that identify and treat depressive symptoms in this population [1, 16]. However, additional mechanism-oriented modeling (informed by directed acyclic graphs) is needed to further understand this relationship. By using lagged covariates, such a study could establish directionality of the depressive symptoms-FI relationship, and explore mediating variables in such a pathway.

Recognizing the exploratory nature of this work, we provide insights into factors that may be targeted for potential intervention. Specifically, given the high levels of injection drug use in co-infected populations [26, 50], substance use treatments and harm reduction programs could potentially reduce FI. A similar potential may exist for mental health services based on the frequent occurrence of depressive symptoms in co-infected individuals [22, 23, 25]. Further research is needed to identify whether the integration of FI screening and FI prevention into these programs is effective at reducing FI and related adverse health outcomes.

### Strengths and Limitations

To our knowledge, our interprovincial study is the first to estimate the prevalence of FI and describe factors associated with FI in an HIV-HCV co-infected population in Canada. Through the use of a validated measure of FI, this work contributes to an evidence base describing FI among a vulnerable subset of the HIV-positive population [51]. It also draws upon longitudinal data from both the FS Study and the CCC, allowing us to explore and describe associations between a variety of factors and FI. Additionally, given minimal non-participation, withdrawals, and losses to follow-up in our study, we do not believe these concerns have biased our results.

There are limitations of this work. First, given the target population of the CCC, the results are most generalizable to co-infected individuals that are currently receiving care in Canada. Also, given that FS Study data collection is ongoing, approximately one-half of the participants only had a baseline measurement as of June 2014. Subsequent analyses of these data will take advantage of longer participant follow-up. Furthermore, by using multiple imputation to address the small proportion of missing data, the non-verifiable assumption that the data were missing at random was made.

Given the sensitive nature of the information collected, there is the potential for measurement error and misclassification of self-reported variables. However, we have no reason to believe that these biases were differential between food secure and food insecure individuals. Also, the ‘Other’ category for province (including participants from Alberta, Saskatchewan, and Nova Scotia) holds no meaningful interpretation given the lack of homogeneity across these provinces. As more data are collected, these provinces will be analyzed separately.

Recent research related to FI has begun to stress the importance of an additional category of FI severity [52, 53], known as marginal FI (some indication of worry or an income-related barrier to adequate food access) [54]. Therefore, instead of being categorized as food secure (0–1 affirmative responses on the HFSSM), a small number of participants (N = 39) could have been categorized in our study as experiencing marginal FI (1 affirmative response) at baseline and as food insecure in the regression models. Therefore, our study underestimates the prevalence of FI in this co-infected population. However, given the small number of participants experiencing marginal FI, we would expect little impact on our conclusions.

It is important to note that the objective was not to estimate the causal effect of one particular factor adjusted for relevant confounders. As such, modeling included a large number of independent variables and multivariate model building was informed by a consideration of statistical significance. Therefore, while insights into potential factors that may be targeted for intervention are provided, our modeling approach does not describe mechanisms whereby these factors act on FI. Also, it is possible that this approach may have resulted in collinearity. For example, education, employment, and income are all socioeconomic factors whose combined effect may be greater than any individual effect, and any individual factors effect may be affected by collinearity. Therefore, conclusions drawn from the study results are best described as hypothesis-generating.

## Conclusions

This study provides evidence regarding sociodemographic, socioeconomic, behavioral, and clinical factors associated with FI in a population of HIV-HCV co-infected individuals. FI is common in this co-infected population and the majority of food insecure participants experienced severe FI. Engagement of co-infected individuals in substance use treatments, harm reduction programs, and mental health services may mitigate FI in this vulnerable subset of the HIV-positive population. Additionally, this work generates hypotheses and provides suggestions for subsequent FI-related research in HIV-positive populations.

## Electronic supplementary material

Below is the link to the electronic supplementary material.

Supplementary material 1 (DOCX 33 kb)
