# Supplementary material for: Food Insecurity in HIV-Hepatitis C Virus Co-infected Individuals in Canada: The Importance of Co-morbidities
Source: AIDS Behav. 2016 Feb 24;21(3):792–802. doi: 10.1007/s10461-016-1326-9 (PMC5306219; doi:10.1007/s10461-016-1326-9)
Supplement: Supplementary file 1 — Supplementary material 1 (DOCX 33 kb) [file 10461_2016_1326_MOESM1_ESM.docx]

**Supplementary Material:**

**Table S1.** Baseline frequency of responses on the 10-item adult scale of the Household Food Security Survey Module (HFSSM) among 525 HIV-HCV co-infected study participants between November 2012 and June 2014, Canada

|  | HFSSM Item | Responses | Frequency (N, %) |
| --- | --- | --- | --- |
| **1** | You and other household members worried that food would run out before you got money to buy more. Was that often true, sometimes true, or never true in the past 6^a^ months? | **Often true**^b^ | 97 (19%) |
|  |  | **Sometimes true** | 190 (36%) |
|  |  | Never true | 238 (45%) |
| **2** | The food that you and other household members bought just didn't last, and there wasn't any money to get more. Was that often true, sometimes true, or never true in the past 6 months? | **Often true** | 95 (18%) |
|  |  | **Sometimes true** | 185 (35%) |
|  |  | Never true | 245 (47%) |
| **3** | You and other household members couldn't afford to eat balanced meals. In the past 6 months was that often true, sometimes true, or never true? | **Often true** | 110 (21%) |
|  |  | **Sometimes true** | 194 (37%) |
|  |  | Never true | 221 (42%) |
| **4** | In the past 6 months, did you or other adults in your household ever cut the size of your meals or skip meals because there wasn't enough money for food? | **Yes** | 209 (40%) |
|  |  | No | 316 (60%) |
| **5** | How often did this happen? *(Referring to Item-4)* | **Almost every month** | 104 (20%) |
|  |  | **Some months but not every month** | 71 (14%) |
|  |  | Only 1 or 2 months | 34 (6%) |
|  |  | Not applicable  *(‘No’ to Item-4)* | 316 (60%) |
| **6** | In the past 6 months, did you ever eat less than you felt you should because there wasn't enough money to buy food? | **Yes** | 204 (39%) |
|  |  | No | 321 (61%) |
| **7** | In the past 6 months, were you ever hungry but didn't eat because you couldn't afford enough food? | **Yes** | 178 (34%) |
|  |  | No | 347 (66%) |
| **8** | In the past 6 months, did you lose weight because you didn't have enough money for food? | **Yes** | 142 (27%) |
|  |  | No | 383 (73%) |
| **9** | In the past 6 months, did you or other adults in your household ever not eat for a whole day because there wasn't enough money for food? | **Yes** | 126 (24%) |
|  |  | No | 399 (76%) |
| **10** | How often did this happen? *(Referring to Item-9)* | **Almost every month** | 50 (9%) |
|  |  | **Some months but not every month** | 55 (11%) |
|  |  | Only 1 or 2 months | 21 (4%) |
|  |  | Not applicable  *(‘No’ to Item-9)* | 399 (76%) |

^a^The HFSSM measures self-reported food insecurity in the past 12 months. Modification of the HFSSM to a shorter reference period, as done in this study (i.e., from 12 to 6 months), has been justified in previous literature.
^b^Responses in **bold** are ‘affirmative responses’ to each item. Health Canada categorizes participants’ food insecurity according to the number of affirmative responses on the HFSSM. Each item can only count as one affirmative response.
